# Feedback-Driven SLAM with Adaptive Point Cloud Selection and Uncertainty-Aware Pose Optimization

**DOI:** 10.3390/s26103275

**Published:** 2026-05-21

**Authors:** Yuqi Shi, Fei Zhang, Zijing Zhang, Ying Hu, Zhanrui Hu

**Affiliations:** 1 College of Automation, Jiangsu University of Science and Technology, Zhenjiang 212100, China; yuqishi@stu.just.edu.cn; 2 Ocean College, Jiangsu University of Science and Technology, Zhenjiang 212100, China; zjhy@just.edu.cn (Y.H.); 232241803818@stu.just.edu.cn (Z.H.); 3 School of Instrument Science and Engineering, Southeast University, Nanjing 210096, China; 230228943@seu.edu.cn

**Keywords:** LiDAR, LiDAR–inertial SLAM, point cloud, closed-loop adjustment

## Abstract

**Highlights:**

**What are the main findings?**

- We develop a LiDAR–inertial SLAM framework where the frontend and backend work in a two-way loop so that backend pose uncertainty and loop information can adjust depth image building and point selection online.
- The method keeps more useful points and uses point quality together with projection error to build point-wise covariance, which is then used in scan-to-map ICP and factor graph optimization to improve pose estimation and map quality.

**What are the implications of the main findings?**

- The proposed system achieved better overall localization results on KITTI and M2DGR, and it also lowered the RMSE in the field test while producing cleaner maps with clearer scene details.
- The ablation results show that feedback control, adaptive point filtering, and covariance-based weighting all help the system stay stable and accurate, especially in long runs and complex scenes.

**Abstract:**

LiDAR SLAM is widely used in robotic navigation and autonomous driving, but many existing methods still handle frontend point cloud processing and backend pose optimization as two loosely connected stages with fixed settings. This can lead to unnecessary computation and also limits the localization performance when the environment or motion changes. To address this issue, we propose a LiDAR–inertial SLAM framework with bidirectional closed-loop coupling between adaptive point cloud processing and pose optimization. In the frontend, depth image resolution is adjusted online according to backend pose uncertainty and loop closure importance, and a comprehensive score integrating point density, depth stability, geometric complexity, and motion consistency is used to select high-quality sparse points. In the backend, the comprehensive score is further combined with depth image quantization error to construct per-point covariance matrices for uncertainty-weighted scan-to-map ICP and factor graph noise modeling. Experiments on the KITTI and M2DGR datasets show that the proposed method reduced the mean RMSE by 15.8% and 15.2%, respectively, compared with FAST-LIO2, while the real-world field test further shows a 26.3% RMSE reduction with respect to the constructed reference trajectory. These results show that the proposed framework improves both mapping quality and localization accuracy.

## 1. Introduction

Simultaneous Localization and Mapping (SLAM) is a key technology for autonomous driving and intelligent robot navigation, and it has received increasing attention with the rapid development of related applications. As a sensor that can stably capture three-dimensional structures, LiDAR plays an important role in SLAM by emitting laser beams and receiving reflected signals to obtain 3D point clouds of the surrounding environment [1]. However, LiDAR usually produces a large amount of point cloud data, and a considerable part of it is redundant. This not only increases computational costs but also introduces noisy observations that may affect the optimization accuracy of SLAM systems [2,3]. In tasks such as large-scale mapping, long-term operation, and multi-robot collaboration, storing and maintaining large point cloud maps also requires considerable memory resources. As a result, many existing systems have to lower the map resolution or reduce the update frequency, which may further influence the localization and mapping performance [4]. Meanwhile, with the improvement in sensor performance and computing platforms, SLAM systems are expected to handle even more data in practical applications, making efficient point cloud processing increasingly important [5].

To reduce computation and storage pressure while improving the usability of point clouds, many studies have introduced point filtering, feature selection, adaptive registration, or uncertainty-aware weighting into LiDAR SLAM and LiDAR–inertial SLAM [6,7,8,9,10]. These methods are effective at reducing data redundancy and improving robustness. However, in many existing pipelines, adaptation is mainly performed within a single stage. Frontend strategies usually decide which points or voxels should be retained according to geometric saliency, environmental structure, or empirical thresholds, while backend strategies mainly adjust residual weights, noise models, or optimization confidence after data association. Therefore, the reliability information estimated by the backend is not always explicitly reused to regulate the next frontend point cloud representation. This separation may limit the ability of the system to maintain a balanced relationship among the point cloud density, observation quality, and pose estimation stability under changing motion and scene conditions.

To address this issue, this paper proposes a feedback-coupled LiDAR–inertial SLAM framework. Rather than treating adaptive point selection, weighted scan-to-map registration, and factor graph optimization as independent components, the proposed method connects them through a closed information route. Backend pose uncertainty and loop closure importance are fed back to regulate depth image resolution and point cloud retention in the next frontend processing stage. At the same time, frontend point quality and projection quantization errors are propagated forward to construct observation covariance weights for scan-to-map iterative closest point (ICP) and factor graph optimization. In this way, the frontend and backend exchange reliability-related information during online operation. The current design is mainly intended for ground mobile robots and vehicle-like platforms, where forward motion and medium-range structural observations are important for stable LiDAR–inertial SLAM. The main contributions of this paper are summarized as follows:

(1) A feedback-coupled frontend–backend regulation mechanism is proposed. Backend pose uncertainty and loop closure importance are compressed into feedback scalars and used to adaptively regulate frontend depth image construction and point cloud retention.
(2) An adaptive depth image construction and point cloud selection strategy is developed. The method combines point density, depth noise, geometric complexity, and motion consistency to retain sparse but informative observations under changing motion and scene conditions.
(3) A frontend-quality-aware covariance weighting strategy is introduced. Point block quality scores are combined with depth image quantization errors to construct observation covariance weights, which are used in weighted scan-to-map ICP and factor graph noise modeling.

## 2. Related Work

This section reviews representative studies related to LiDAR-only SLAM, LiDAR–inertial odometry and mapping, adaptive and uncertainty-aware estimation, and learning-based LiDAR SLAM. These works provide the technical background for point cloud selection, scan-to-map registration, backend optimization, and uncertainty modeling in the proposed framework.

### 2.1. LiDAR-Only and Loosely Coupled LiDAR–Inertial SLAM

Early LiDAR SLAM methods mainly estimated motion from geometric features or scan registration, providing the foundation for many later LiDAR–inertial systems. LOAM [11] decomposes LiDAR odometry and mapping into two parallel modules and uses edge and planar features to reduce computation, but it remains sensitive to feature degradation and accumulated drift. LeGO-LOAM [12] further introduces ground segmentation and lightweight optimization for ground vehicles, while surfel-based SLAM and SC-F-LOAM improve data association, map representation, and loop correction for large-scale environments [13,14]. These methods are effective and efficient in structured scenes, but their performance can still be affected by fast motion, sparse geometric structures, dynamic interference, and long-distance drift.

Recent LiDAR-only odometry methods have shown that simple registration pipelines can still achieve a strong performance when carefully designed. KISS-ICP improves the robustness of point-to-point ICP through motion compensation, scan subsampling, and adaptive thresholding [6]. GenZ-ICP further adjusts registration weights according to environmental geometric characteristics, improving robustness in degenerate scenes [7]. In addition, some loosely coupled frameworks incorporate external absolute constraints to improve global consistency. For example, PPP/INS/LiDAR SLAM combines LiDAR–inertial odometry with PPP information through graph optimization [15]. However, these methods mainly focus on LiDAR-only registration robustness or loosely coupled external correction, and their performance still depends on the registration quality and the availability of reliable external constraints.

### 2.2. LiDAR–Inertial Odometry and Mapping

LiDAR–inertial odometry and mapping has become a mainstream solution because LiDAR provides accurate geometric constraints while inertial measurement unit (IMU) measurements provide high-frequency motion prediction and motion compensation. LIO-SAM [16] formulates LiDAR–IMU fusion as a factor graph and integrates IMU preintegration, LiDAR odometry, GPS factors, and loop closure to improve global consistency. Although loop closure is included in LIO-SAM as a global constraint, the loop-related state is not explicitly fed back to regulate the next frontend point cloud representation. LVI-SAM [17] further incorporates visual information to enhance robustness through a LiDAR–visual–inertial framework. Other methods introduce scene-specific constraints or external observations to improve stability in degraded or large-scale environments, such as dynamic coplanarity constraints, IMU-centric processing, LiDAR–inertial–GNSS fusion, and multi-platform collaborative mapping [18,19,20,21,22]. These methods improve robustness from different perspectives, but their performance can be affected by feature quality, data association, sensor synchronization, and the reliability of loop closure or external constraints.

Filtering-based and direct LiDAR–inertial methods emphasize real-time state estimation and efficient scan-to-map updates. FAST-LIO2 [23] directly registers raw points to an incremental map through an iterated Kalman filter, avoiding explicit feature extraction and achieving high efficiency. DLIO [24] is a lightweight direct LiDAR–inertial odometry framework with continuous-time motion correction, while Point-LIO [25] performs point-wise LiDAR–inertial updates for high-bandwidth state estimation. These methods demonstrate the advantages of tightly coupled direct fusion, especially in real-time odometry. However, their frontend point representation is generally not explicitly adjusted according to the posterior pose uncertainty or loop closure state. Therefore, when scene structure changes or geometric constraints become weak, the retained observations and backend uncertainty handling may not be coordinated in a closed manner.

Map representation and residual modeling have also been studied to improve scan-to-map robustness. LIO-GVM [26] introduces Gaussian voxel maps and incorporates variance information into residual metrics, while VINA-SLAM [27] uses voxel-based mapping, normal-guided correspondence, and planar regularization to improve LiDAR–IMU registration in degenerate scenes. These methods show the value of map-level statistical information and geometric constraints, but their performance still depends on the voxel or map statistics, correspondence quality, and parameter settings in degenerate or dynamic scenes.

### 2.3. Adaptive and Uncertainty-Aware LiDAR–Inertial Methods

Adaptive processing and uncertainty modeling are closely related to the proposed method. Adaptive-LIO adjusts segmentation, motion handling, and map resolution according to environmental changes [9]. LOG-LIO2 models LiDAR measurement uncertainty and surface-related uncertainty to improve uncertainty propagation in LiDAR–inertial odometry [8]. UA-LIO further introduces uncertainty-aware modeling for autonomous driving in urban environments [10]. These studies show that environmental adaptation and uncertainty-aware estimation are useful for improving robustness and accuracy in LiDAR–inertial systems.

Compared with these methods, the proposed framework focuses on the closed exchange of reliability-related information between the frontend and backend. Backend pose uncertainty and loop closure importance are used to regulate the next depth image resolution and point cloud retention, while frontend point quality and projection quantization errors are propagated forward to construct observation covariance weights for scan-to-map ICP and factor graph optimization. Accordingly, this work does not treat adaptive filtering or uncertainty weighting as isolated techniques but organizes them into a feedback-coupled LiDAR–inertial SLAM pipeline.

### 2.4. Learning-Based LiDAR SLAM

Learning-based methods have also been introduced into LiDAR SLAM to improve scene representation, loop closure, and semantic understanding. SNI-SLAM++ and Loopy-SLAM use neural implicit or dense neural representations to enhance mapping and loop correction [28,29]. Semantic LiDAR SLAM methods, such as SuMa++, use semantic segmentation to filter dynamic objects and assist matching [30]. PIN-SLAM further adopts point-based implicit neural representations to improve global map consistency without explicit correspondence searching [31]. Other works embed online learning modules into factor graph optimization to adapt to motion errors and improve stability in degraded scenes [32]. Although these methods improve scene understanding and map consistency, they often require additional training, semantic inference, or neural optimization. In contrast, the proposed method improves adaptability through explicit frontend–backend feedback regulation and reliability-aware optimization within a conventional LiDAR–inertial SLAM framework, where unstable observations are suppressed through point quality evaluation, covariance weighting, and robust residual weighting, reducing reliance on training-based semantic inference or neural map optimization.

## 3. Method

**Figure 1:**
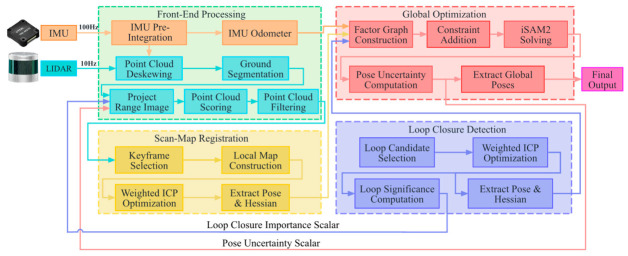
The overall flowchart.

The proposed method is designed as a feedback-coupled LiDAR–inertial SLAM pipeline. Its key idea is the explicit exchange of reliability-related variables between frontend point cloud processing and backend pose optimization. The backend estimates the pose uncertainty and loop closure importance, which are fed back to regulate the depth image resolution and point cloud selection, while the frontend assigns quality scores to retained point blocks and combines them with projection quantization errors to construct point-wise covariance weights for scan-to-map ICP and factor graph optimization. Thus, point cloud regulation and backend uncertainty weighting are organized as a closed information route. The overall flowchart is shown in Figure 1.

### 3.1. Establishment of Adaptive Depth Image

We first project the point cloud onto a depth image to enable subsequent computation. Traditional depth image generation usually relies on fixed resolutions and uniform angular grids, which may not balance computational efficiency and geometric fidelity under changing motion or scene conditions. To address this issue, we use a practical adaptive depth image construction strategy. The purpose is not to derive a globally optimal image resolution but to increase angular representation when the backend indicates unreliable pose estimation or an important loop closure window, and to reduce redundant representation when the system is stable.

The input LiDAR scan is first deskewed using IMU preintegration, and ground points are removed with an existing filtering method. The remaining non-ground points are then converted from the Cartesian coordinate system (x,y,z) to the spherical coordinate system (r,θ,ϕ) according to the following formulas:

(1) $$\left\{\begin{array}{l} r=\sqrt{x^{2}+y^{2}+z^{2}}\\ \phi =\arcsin\left(\frac{z}{r}\right)\\ \theta =\mathrm{atan}2(y,x) \end{array}\right.$$

where r represents the distance to the LiDAR, corresponding to the depth value. ϕ and θ denote the elevation and azimuth angles of the spherical coordinate system, corresponding to the depth image’s vertical and horizontal angles, respectively.

Then, to adapt to SLAM backend state changes, we construct an adaptive partitioning rule for point cloud mapping. The resolution is adjusted based on the pose uncertainty scalar from the previous optimization step ($\mu_{t-1}$) and the loop closure importance scalar from the previous optimization step ($l_{t-1}$). Letting H and W denote the vertical and horizontal resolutions, the resolution adjustment coefficient (κ) is calculated as:

(2) $$\kappa =\max(\min(1+k_{u}u_{t-1}+k_{l}l_{t-1},\kappa_{\max}),\kappa_{\min})$$

where $\kappa_{\min}$ and $\kappa_{\max}$ represent the lower and upper bounds of the adjustment coefficient, respectively, while $k_{u}$ and $k_{l}$ are weight coefficients. The pose uncertainty scalar (μ) indicates the need for denser observations when pose estimation becomes less reliable, while the loop closure importance scalar (l) helps preserve structural information during loop verification. The bounds of κ prevent excessive downsampling or computational growth; thus, κ serves as a lightweight reliability-driven resolution controller.

Based on the preset base resolution ($H_{\mathrm{base}}\times W_{\mathrm{base}}$), the resolution of the current frame H×W is obtained via the following formulas:

(3) $$H=\left\lceil H_{\mathrm{base}}\kappa \right\rceil$$

(4) $$W=\left\lceil W_{\mathrm{base}}\kappa \right\rceil$$

**Figure 2:**
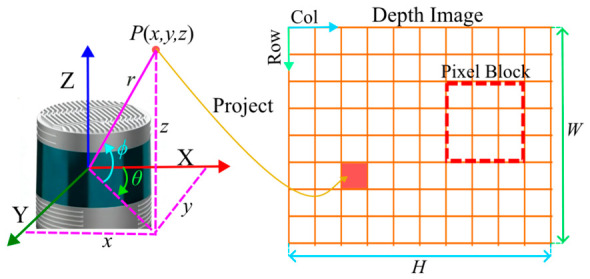
Schematic diagram of depth image construction for proposed method.

After defining the depth image resolution, each 3D point is projected onto the 2D pixel grid according to its spherical coordinates. For vertical discretization, the median elevation angle (ϕ) of the points assigned to each row is used as the representative row angle. For horizontal discretization, the mean azimuth angle (θ) of the points assigned to each column is used as the representative column angle. Pixels receiving projected points are marked as valid pixels, and the point whose depth is closest to the pixel-average depth is selected as the reference point. The valid pixels are then grouped into small pixel blocks according to preset angular intervals, as shown in Figure 2.

### 3.2. High-Quality Point Cloud Filtering

**Figure 3:**
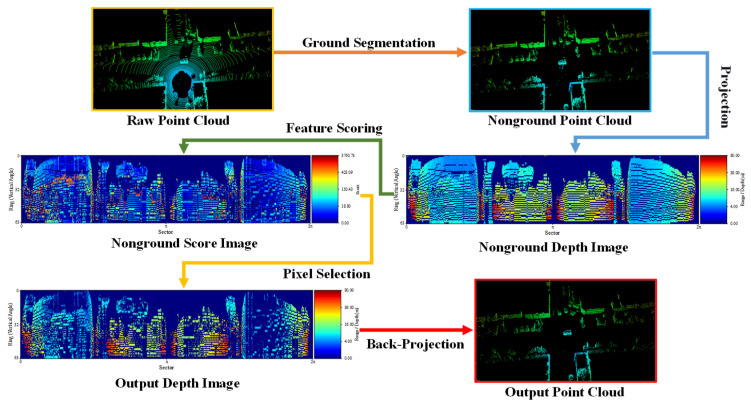
Flowchart of process for filtering high-quality point clouds.

Based on the adaptive depth image, four lightweight metrics are calculated for each pixel block to describe the observation usefulness: the sampling sufficiency, depth stability, geometric constraint strength, and temporal visibility. These metrics are fused into a comprehensive score to select sparse but informative point clouds. The filtering process is shown in Figure 3, and the detailed calculation is given below.

(1) First, the feature evaluation metric regarding the point cloud density within each pixel block is calculated as follows:

Let u and v denote the column and row indices of any valid pixel, respectively, while s and h represent the indices of any pixel block. The spatial coverage area of a valid pixel (u,v) is defined as A(u,v), calculated using the following formula:

(5) $$A(u,v)=r{\left(u,v\right)}^{2}\times \Delta \theta \times \Delta \phi$$

where r(u,v) represents the depth value of the valid pixel, while Δθ and Δϕ denote the angular spans of the valid pixel in the horizontal and vertical directions, respectively.

The spatial coverage area of a pixel block (s,h) is defined as $A_{\mathrm{block}}(s,h)$, calculated as:

(6) $$A_{\mathrm{block}}(s,h)=\sum_{\left(u,v)\in (s,h\right)}A(u,v)$$

Letting N(s,h) be the count of valid pixels within the pixel block (s,h), the point cloud density (ρ(s,h)) within this block is calculated as follows:

(7) $$\rho (s,h)=\frac{N(s,h)}{A_{\mathrm{block}}(s,h)}$$

Subsequently, ρ(s,h) is normalized to obtain the feature evaluation metric for the point cloud density, denoted as ${\hat{f}}_{1}(s,h)$ for each pixel block.

(2) Subsequently, the feature evaluation metric for the depth value noise within each pixel block is calculated as follows:

The depth values of all valid pixels within the pixel block (s,h) are extracted to form a sample set, and their median is calculated and denoted as $r_{\mathrm{med}}(s,h)$. Then, the Median Absolute Deviation (MAD) is employed to measure the dispersion degree of depth values in that region, thereby eliminating the influence of individual noise points on the overall evaluation. The specific calculation formula is as follows:

(8) $${\mathrm{MAD}}_{r}(s,h)={\mathrm{median}}_{\left(u,v)\in (s,h\right)}|r(u,v)-r_{\mathrm{med}}(s,h)|$$

Finally, the calculated ${\mathrm{MAD}}_{r}(s,h)$ is normalized and inversely mapped to obtain the feature evaluation metric (${\hat{f}}_{2}(s,h)$), which reflects the degree of local depth value noise.

(3) Afterwards, the feature evaluation metric for geometric complexity within each pixel block is calculated as follows:

To efficiently capture edge and slope features, first-order gradients are employed to measure local geometric variations. For each valid pixel within the pixel block (s,h) the central difference method estimates the horizontal gradient ($g_{\theta }(u,v)$) and vertical gradient ($g_{\phi }(u,v)$) utilizing adjacent depth values. Subsequently, the arithmetic mean of the gradient magnitudes of all valid pixels is calculated as the feature evaluation metric for geometric complexity, formulated as follows:

(9) $$\mathrm{Curv}(s,h)=\frac{1}{N(s,h)}\sum_{\left(u,v)\in (s,h\right)}\sqrt{g_{\theta }{\left(u,v\right)}^{2}+g_{\phi }{\left(u,v\right)}^{2}}$$

This metric is normalized to obtain the metric score for geometric complexity (${\hat{f}}_{3}(s,h)$).

(4) Finally, the feature evaluation metric for motion perception is calculated to optimize SLAM frontend processing by identifying high-value forward regions.

Given the relative pose from the current frame to the latest keyframe, the translational vector ($t_{\mathrm{cur}}^{\mathrm{key}}$) is normalized to extract the motion direction (${\hat{v}}_{\mathrm{cur}}^{\mathrm{key}}$).

Simultaneously, the central line-of-sight direction of the pixel block ((s,h)) is defined as $d^{\left(\mathrm{cur}\right)}(s,h)$, calculated as follows:

(10) $$d^{\left(\mathrm{cur}\right)}(s,h)=\left[\begin{array}{c} \cos\bar{\phi }(s,h)\cos\bar{\theta }(s,h)\\ \cos\bar{\phi }(s,h)\sin\bar{\theta }(s,h)\\ \sin\bar{\phi }(s,h) \end{array}\right]$$

where $\bar{\theta }(s,h)$ and $\bar{\phi }(s,h)$ denote the mean representative azimuth and elevation angles, respectively, of the columns and rows covered by the pixel block ((s,h)), thereby characterizing its horizontal and vertical central viewing directions.

Subsequently, $d^{\left(\mathrm{cur}\right)}(s,h)$ is transformed to the keyframe coordinate system and normalized to obtain ${\hat{d}}^{\left(\mathrm{key}\right)}(s,h)$.

The cosine alignment (η(s,h)) between the line-of-sight and motion direction is then calculated to measure the region’s consistency with the platform motion:

(11) $$\eta (s,h)={\hat{d}}^{\left(\mathrm{key}\right)}{\left(s,h\right)}^{⊤}{\hat{v}}_{\mathrm{cur}}^{\mathrm{key}}\in [-1,1]$$

Forward-view regions usually remain visible for longer and provide more temporally consistent observations under ground-vehicle motion. Therefore, an exponent (*γ*) is introduced as a focusing factor to suppress non-forward regions:

(12) $$w_{\mathrm{rel}}(s,h)=\max{\left(0,\eta (s,h)\right)}^{\gamma }$$

Finally, a preset band-pass weight ($w_{\mathrm{range}}(r)$) is defined to prioritize medium distances consistent with the SLAM algorithm’s optimal observation range. Incorporating $w_{\mathrm{range}}(r)$, the final motion perception score (M(s,h)) is obtained as follows:

(13) $$M(s,h)=w_{\mathrm{rel}}(s,h)\cdot w_{\mathrm{range}}(r)$$

Based on the final motion perception score, the result is normalized to yield ${\hat{f}}_{4}(s,h)$.

Together, these four metrics provide a lightweight description of the local observation usefulness from the aspects of sampling density, range stability, geometric variation, and motion consistency. They are used as practical cues for point selection and are then fused with backend state feedback to obtain the final comprehensive feature score.

Based on the aforementioned four feature evaluation metrics, ${\hat{f}}_{1}(s,h)$ to ${\hat{f}}_{4}(s,h)$, a basic comprehensive feature score is calculated as follows:

(14) $$U_{\mathrm{geom}}(s,h)=\sum_{i=1}^{4}w_{i}^{\mathrm{base}}{\hat{f}}_{i}(s,h)$$

where $w_{i}^{\mathrm{base}}$ represents the weight corresponding to the i−th feature metric.

Subsequently, two supplementary incentive terms are defined: The degeneration term ($U_{\deg}(s,h)={\hat{f}}_{1}(s,h)+{\hat{f}}_{3}(s,h)$) retains sparse and edge features to stabilize pose estimation under high uncertainty. The loop closure term ($U_{\mathrm{loop}}(s,h)={\hat{f}}_{3}(s,h)+{\hat{f}}_{4}(s,h)$) preserves structural and motion-consistent regions for loop closure optimization. Finally, the comprehensive feature score ($U^{\mathrm{fused}}(s,h)$) is calculated as follows:

(15) $$U^{\mathrm{fused}}(s,h)=U_{\mathrm{geom}}(s,h)+\lambda_{u}\mu U_{\deg}(s,h)+\lambda_{l}lU_{\mathrm{loop}}(s,h)$$

where $\lambda_{u}$ and $\lambda_{l}$ regulate the term proportions. The score is normalized within the range of [0,1] to yield $\tilde{U}(s,h)$. Pixel blocks satisfying $\tilde{U}(s,h)>U_{\mathrm{th}}$ are retained, while others are discarded.

**Figure 4:**
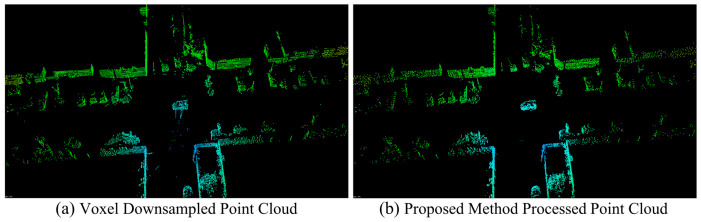
Comparison of point clouds obtained by uniform downsampling and our proposed method.

Finally, only the reference point of each retained pixel is mapped back to 3D Cartesian space via inverse spherical transformation, while all other points within the pixel are discarded, yielding the final high-quality sparse point cloud. Figure 4 compares the point clouds obtained via uniform downsampling and our proposed method. Both retain only about 20% of the original non-ground points.

As shown in Figure 4, compared with uniform downsampling, the proposed method exhibits two desirable characteristics: it produces a much cleaner point cloud with very few noisy or isolated outlier points, and it preserves more points in near-range and forward-view regions while suppressing less informative far-range points. This non-uniform retention pattern is more consistent with the reliability of LiDAR observations and the requirements of downstream SLAM optimization.

It should be noted that the weights and thresholds are empirically chosen and fixed across all sequences, with frame-wise normalization applied to reduce metric scale differences. The score is not a universal optimal rule; it is designed for ground vehicles where forward-view structures dominate scan-to-map constraints. For different LiDAR beam patterns or non-forward-dominant platforms, the weights and motion perception term may need recalibration.

### 3.3. Quantization Error Modeling and Covariance Estimation

Upon obtaining the filtered point cloud, we construct an approximate reliability covariance for each retained point. This covariance is not intended to be a fully calibrated sensor noise model. Instead, it provides a relative confidence measure for backend optimization by combining two factors: projection quantization error caused by depth image compression and the frontend quality score of the corresponding pixel block. Points with larger projection uncertainty or lower quality scores are assigned larger variances and therefore smaller optimization weights.

**Figure 5:**
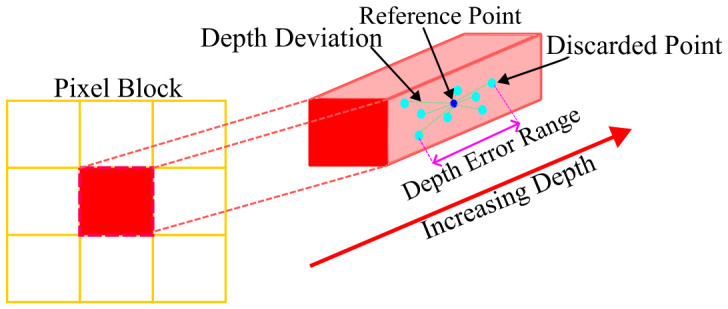
A schematic of range quantization error caused by pixel-wise depth compression. Multiple 3D points with different depths falling within the same pixel are represented by a single reference point, resulting in depth deviations along the viewing ray.

**Figure 6:**
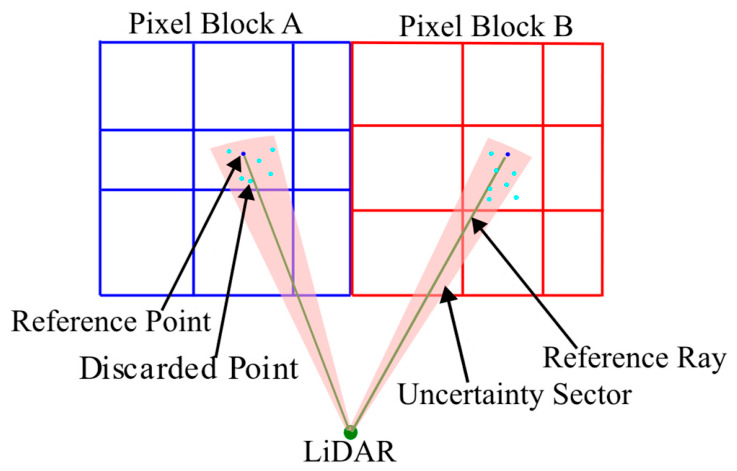
A schematic of angular quantization error caused by pixel-wise directional approximation. Discarded points within a pixel are represented by a single reference ray, producing an angular uncertainty sector after pixel projection. Only one angular dimension is illustrated for clarity.

We first analyze the errors introduced during the aforementioned point cloud filtering process, which primarily yields two types of quantization errors: (1) range quantization error, occurring when multiple 3D points with different ranges fall into the same pixel and are represented by a single depth value; and (2) angular quantization error, introduced because all rays covered by the pixel are approximated by one representative direction in both the horizontal and vertical dimensions. The schematics of these two errors are shown in Figure 5 and Figure 6.

To better quantitatively describe these errors, we define: $r_{q}$ as the difference between the maximum and minimum depth values in a valid pixel; $\Delta \theta_{q}$ as the average horizontal angular difference between all discarded points and the reference point within the same pixel; and $\Delta \phi_{q}$ as the corresponding average vertical angular difference between the discarded points and the reference point. These quantities characterize the local dispersion introduced by pixel-wise depth image compression and are used as reliability cues for the retained reference point rather than as independently calibrated sensor noise measurements.

Subsequently, we incorporate the comprehensive feature score corresponding to each pixel to model the obtained range and angular quantization errors, as follows:

(16) $$\sigma_{r}^{2}=\frac{r_{q}^{2}}{12}+\alpha_{r}^{2}\cdot {\left(1-\tilde{U}\right)}^{2}$$

(17) $$\sigma_{\theta }^{2}=\frac{\Delta \theta_{q}^{2}}{12}+\beta_{\theta }^{2}\cdot {\left(1-\tilde{U}\right)}^{2}$$

(18) $$\sigma_{\phi }^{2}=\frac{\Delta \phi_{q}^{2}}{12}+\beta_{\phi }^{2}\cdot {\left(1-\tilde{U}\right)}^{2}$$

where $\alpha_{r}^{2}$, $\beta_{\theta }^{2}$ and $\beta_{\phi }^{2}$ are the parameters regulating the proportion of the comprehensive feature score, and $\tilde{U}$ is the comprehensive feature score of the corresponding pixel block for each pixel.

By integrating the three aforementioned components, we construct the diagonal quantization error covariance matrix ($\Sigma_{r\theta \phi }$) in the spherical coordinate system:

(19) $$\Sigma_{r\theta \phi }=\left[\begin{array}{ccc} \sigma_{r}^{2} & 0 & 0\\ 0 & \sigma_{\theta }^{2} & 0\\ 0 & 0 & \sigma_{\phi }^{2} \end{array}\right]$$

Finally, using the Jacobian matrix ($J_{\mathrm{cart}}$), we propagate $\Sigma_{r\theta \phi }$ to 3D Cartesian space to obtain the observation covariance matrix ($\Sigma_{xyz}$), which provides weights for backend optimization.

### 3.4. ICP Weighted Optimization of Scan-to-Map

To enhance backend stability, we perform scan-to-map weighted ICP registration utilizing the obtained high-quality point clouds and covariance matrices to estimate the relative poses between frames.

To construct a consistent local map, keyframes are selected by jointly considering sensor motion and depth image information gain. For the current frame (t), the planar displacement ($d_{t}$) and heading change ($\Delta \psi_{t}$) are computed relative to the last keyframe. The overlap ratio ($o_{t}$) is calculated from the valid-pixel overlap after projecting the current point cloud into the previous keyframe coordinate system. Within the overlapping region, pixels whose point counts change beyond a preset threshold are treated as information-rich pixels, and their proportion defines the gain ratio ($g_{t}$). These terms are fused into the keyframe selection score ($K_{t}$):

(20) $$K_{t}=\frac{g_{t}+\beta_{1}d_{t}+\beta_{2}\Delta \psi_{t}}{o_{t}+\epsilon }$$

where $\beta_{1}$ and $\beta_{2}$ are weighting coefficients for the pose changes, and ϵ is a small constant to prevent division by zero. If $K_{t}>K_{\mathrm{th}}$, the current frame is saved as a keyframe.

Compared with simple time or motion threshold rules and more complex uncertainty- or entropy-based keyframe selection strategies, the proposed criterion adopts a lightweight hybrid design. It jointly considers pose variation, valid-pixel overlap, and depth image information gain so that informative frames can be retained while redundant local observations are suppressed. This design is consistent with the proposed depth image frontend and can be computed online with little additional cost.

After the keyframes are generated, we employ a spatiotemporal selection strategy. Temporally, candidate keyframes are selected from the last 10 s (extended to 20 s if insufficient). Spatially, these candidates are further filtered based on their planar Euclidean distances to the current frame, and the closest ones are fused to form the final local map.

For the i−th point in the current frame, denoted as the source point ($p_{\mathrm{src},i}$), the geometric residual ($e_{i}$) between this point transformed into the local map frame and the corresponding target plane is:

(21) $$e_{i}=n_{i}^{⊤}(T(\xi )p_{\mathrm{src},i}-p_{\mathrm{map},i})$$

where ξ is the Lie algebra variable representing the transformation, T(ξ) denotes the rigid body transformation matrix to be estimated from the current frame to the map frame, $p_{\mathrm{map},i}$ is the corresponding point found in the local map point cloud with the closest Euclidean distance to the transformed source point, and $n_{i}$ is the local surface normal vector at $p_{\mathrm{map},i}$.

To enhance accuracy, $\Sigma_{xyz,i}$ is transformed to the local map frame via the rotation matrix ($R_{\mathrm{src}}^{\mathrm{map}}$) and projected along the target normal ($n_{i}$) to calculate the residual variance. Consequently, the covariance weight ($w_{\mathrm{cov},i}$) is constructed as:

(22) $$w_{\mathrm{cov},i}=\frac{1}{n_{i}^{⊤}R_{\mathrm{src}}^{\mathrm{map}}\Sigma_{xyz,i}{\left(R_{\mathrm{src}}^{\mathrm{map}}\right)}^{⊤}n_{i}+\varsigma }$$

where ς is a small constant used to prevent division by zero. This projection assigns a smaller weight to observations with larger uncertainty along the target normal direction, thereby reducing their influence in the subsequent ICP update.

In the actual optimization, this weight is combined with the Huber kernel weight ($w_{\mathrm{robust}}$) to form the final total weight, which is substituted into the normal equations of the Levenberg–Marquardt (LM) to construct the Hessian matrix:

(23) $$H=\sum_{i}w_{\mathrm{cov},i}\cdot w_{\mathrm{robust}}J_{i}^{⊤}J_{i}$$

where $J_{i}$ is the Jacobian matrix of the residual with respect to the Lie algebra variable. The Huber kernel is used as a standard robust term in scan-to-map optimization to reduce the influence of large residuals caused by mismatches, occlusions, moving objects, and local degeneracy [33,34,35]. Here, $\delta_{\mathrm{Huber}}$ denotes the Huber threshold that controls the transition from quadratic to linear residual weighting. Compared with Cauchy and Tukey kernels, Huber uses a relatively mild down-weighting strategy, which helps preserve useful geometric constraints while complementing the proposed covariance-based weighting.

Consequently, the ICP algorithm iteratively estimates the optimal rigid body transformation matrix (T(ξ)) from the current frame to the map frame. Meanwhile, the Hessian matrix at convergence is passed to the subsequent factor graph for adaptive noise model settings.

Since the local map is constructed from previously optimized historical keyframes and treated as fixed during the current update, the converged scan-to-map result is used as a LiDAR pose constraint for the current keyframe in the factor graph, with covariance derived from the corresponding Hessian.

### 3.5. Loop Closure Detection

To detect loop closures, temporally adjacent keyframes are first excluded to avoid trivial matches. The remaining historical keyframes are coarsely filtered using the planar displacement ($d_{t}$) and heading change ($\Delta \psi_{t}$) and are then evaluated by spatial overlap and coarse geometric consistency. The candidate with sufficiently large overlap and the best matching potential is selected as the loop frame. A weighted point-to-plane ICP is then performed between the current frame and the selected loop frame, and the resulting relative pose and Hessian matrix are passed to the backend factor graph.

The loop closure importance scalar (l), which is fed back to the frontend point cloud processing, is computed from the geometric overlap and ICP fitting quality. We first define an instantaneous loop score ($\tilde{l}$). If a valid loop candidate is detected, $\tilde{l}=0.5(s_{\mathrm{loop}}+s_{\mathrm{icp}})$, where $s_{\mathrm{loop}}$ is the overlap score defined in Section 3.4, and $s_{\mathrm{icp}}$ is obtained by normalizing the inverse RMS distance of inlier correspondences after ICP convergence. If no valid loop is detected, $\tilde{l}=0$.

To reduce abrupt changes, temporal exponential smoothing is applied:

(24) $$l=(1-\tau_{\mathrm{loop}})l_{t-1}+\tau_{\mathrm{loop}}\tilde{l}$$

where $\tau_{\mathrm{loop}}$ is the temporal smoothing coefficient, and $l_{t-1}$ is the previous scalar. l rises rapidly upon loop occurrence and decays slowly, sustaining improved frontend quality during the critical loop closure window.

### 3.6. Global Optimization

**Figure 7:**
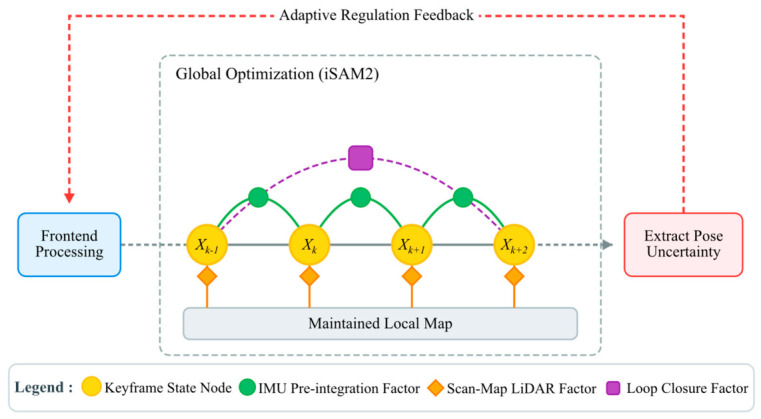
Illustration of pose graph structure for global optimization.

To ensure global consistency, we integrate the odometry and loop closure modules into a factor graph framework. The optimized pose covariance is then extracted to provide feedback for frontend regulation, as shown in Figure 7.

The state variable for the k-th keyframe is defined as $X_{k}=\{X_{k},V_{k},B_{k}\}$. Here, $X_{k}\in SE(3)$ denotes the absolute pose in the world coordinate system; $V_{k}\in {\mathbb{R}}^{3}$ represents the velocity vector in 3D space; and $B_{k}=\{b_{g,k},b_{a,k}\}$ denotes the IMU gyroscope and accelerometer biases. In implementation, the first keyframe state is fixed as the global reference during optimization, which removes the gauge freedom of the factor graph. The global cost function, defined as the weighted sum of squared residuals, is calculated as follows:

(25) $$\min_{\left\{X_{k}\right\}}\sum_{k}\|e_{\mathrm{lidar},k}{\|}_{\Sigma_{\mathrm{lidar},k}^{-1}}^{2}+\sum_{k}\|e_{\mathrm{imu},k}{\|}_{\Sigma_{\mathrm{imu},k}^{-1}}^{2}+\sum \|e_{\mathrm{loop}}{\|}_{\Sigma_{\mathrm{loop}}^{-1}}^{2}$$

where $\|\eta \;{\|}_{W}^{2}=\eta^{T}W\eta$ denotes the weighted squared norm. The residual terms $e_{\mathrm{lidar},k}$, $e_{\mathrm{imu},k}$, and $e_{\mathrm{loop}}$ represent the relative transformation errors derived from LiDAR odometry, IMU preintegration constraints, and loop closure detection, respectively.

Unlike the IMU factor, whose covariance matrix ($\Sigma_{\mathrm{imu},k}$) is intrinsically determined by the hardware noise characteristics of the sensor, the covariances for the LiDAR and loop closure factors are dynamically evaluated. Specifically, for the LiDAR odometry factor, the covariance matrix ($\Sigma_{\mathrm{lidar},k}$) is derived from the Hessian matrix ($H_{k}$) obtained at the convergence of the scan-to-map weighted ICP:

(26) $$\Sigma_{\mathrm{lidar},k}={\left(H_{k}+I\right)}^{-1}$$

where I is the identity matrix. Similarly, for the loop closure factor, the covariance matrix ($\Sigma_{\mathrm{loop}}$) is also modeled using the Hessian matrix at the convergence of the loop detection ICP:

(27) $$\Sigma_{\mathrm{loop}}={\left(H_{\mathrm{loop}}+I\right)}^{-1}$$

Here, the inverse Hessian provides a local approximation of the registration uncertainty around the converged ICP solution, allowing the LiDAR odometry and loop closure factors to be assigned confidence levels according to their local constraint strength.

We formulate the global cost function as a factor graph and solve it using Incremental Smoothing and Mapping 2 (iSAM2) [36]. Upon adding new keyframes or constraints, iSAM2 performs local linearization and incremental updates only on affected variables, ensuring online efficiency. Ultimately, this yields the optimal state variable.

To realize closed-loop coupling between the frontend and backend, we extract the pose uncertainty of the latest keyframe from the optimizer and compress it into a pose uncertainty scalar. Let $\Sigma_{k}^{\mathrm{pose}}\in {\mathbb{R}}^{6\times 6}$ be the covariance matrix of the latest keyframe pose under Lie algebra perturbation. Focusing on planar stability, we extract the planar translation covariance submatrix ($\Sigma_{k}^{\mathrm{xy}}$) and yaw variance ($\sigma_{\psi ,k}^{2}$) from $\Sigma_{k}^{\mathrm{pose}}$. By calculating the square root of the trace of $\Sigma_{k}^{xy}$ and the square root of $\sigma_{\psi ,k}^{2}$ and normalizing the results using empirical thresholds, we obtain the normalized translation uncertainty ($\mu_{k}^{p}$) and yaw uncertainty ($\mu_{k}^{\psi }$). These are then weighted to yield μ:

(28) $$\mu =\lambda_{p}\mu_{k}^{p}+\lambda_{\psi }\mu_{k}^{\psi }$$

where $\lambda_{p}$ and $\lambda_{\psi }$ are weights. μ serves as feedback for the adaptive fusion of comprehensive feature scores in the frontend, increasing the focus on critical regions during system instability.

Based on the methodology described above, the overall flow of the algorithm is presented in Algorithm 1.
**Algorithm 1:** Bidirectional closed-loop LiDAR–inertial SLAM**Input:** $\left\{P_{t}\right\}$: LiDAR scans; $H_{\mathrm{base}}\times W_{\mathrm{base}}$: base depth-image resolution; $U_{th}$: block-retention threshold; $K_{\mathrm{th}}$: keyframe threshold; Θ: fixed coefficients. **Output: **$\left\{X_{k}\right\}$: optimized keyframe states, $X_{k}=\;\left\{X_{k},V_{k},B_{k}\right\}$; $\mu_{t}$: pose-uncertainty scalar; $l_{t}$: loop-closure importance scalar.
1:       $G\leftarrow \;\emptyset ,\;K\leftarrow \;\emptyset ,\;\mu_{0}\leftarrow \;0,\;l_{0}\leftarrow \;0$
2:       **for** each scan $P_{t}$ **do**
3:              deskew $P_{t}$ and remove ground points
4:              compute spherical coordinates $\left(r,\theta ,\varphi \right)$
5:              $\kappa_{t}\leftarrow \;\mathrm{clamp}\left(1\;+k_{u}\mu_{t-1}+k_{l}l_{t-1},\kappa_{\min},\kappa_{\max}\right)$
6:              $H_{t}\leftarrow \;\left\lceil H_{\mathrm{base}}\kappa_{t}\right\rceil ,\;W_{t}\leftarrow \;\left\lceil W_{\mathrm{base}}\kappa_{t}\right\rceil$
7:              $D_{t}\leftarrow$ project $P_{t}$ onto a $H_{t}\times W_{t}$ depth image
8:              $\Omega_{t}\leftarrow$ partition $D_{t}$ into pixel blocks $\left\{\left(s,h\right)\right\}$
9:              **for** each block $\left(s,h\right)\;\in \Omega_{t}$ **do**
10:                    compute $\rho \left(s,h\right),\;\mathrm{MADr}\left(s,h\right),\;\mathrm{Curv}\left(s,h\right),M\left(s,h\right)$ by Equations (5)–(13)
11:                    normalize to ${\hat{f}}_{1},\;{\hat{f}}_{2},\;{\hat{f}}_{3},\;{\hat{f}}_{4}$
12:                    compute $U_{\mathrm{geom}}\left(s,h\right)\;\leftarrow \sum {w_{i}}^{\mathrm{base}}{\hat{f}}_{i}\left(s,h\right)$
13:                    $U_{\deg}\left(s,h\right)\;\leftarrow \;{\hat{f}}_{1}\left(s,h\right)\;+{\hat{f}}_{3}\left(s,h\right)$
14:                    $U_{\mathrm{loop}}\left(s,h\right)\;\leftarrow {\hat{f}}_{3}\left(s,h\right)\;+{\hat{f}}_{4}\left(s,h\right)$
15:                    $U_{\mathrm{fused}}\left(s,h\right)\;\leftarrow U_{\mathrm{geom}}\left(s,h\right)\;+\lambda_{u}\mu_{t-1}U_{\deg}\left(s,h\right)\;+\lambda_{l}l_{t-1}U_{\mathrm{loop}}\left(s,h\right)$
16:                    normalize $U_{\mathrm{fused}}\left(s,h\right)$ to $U\left(s,h\right)$
17:              **end for**
18:              retain ${p_{t}}^{\mathrm{ref}}\left(u,v\right)$ **if**
$U\left(s,h\right)\;>U_{\mathrm{th}}$; form ${P_{t}}^{\mathrm{sel}}$
19:              ${\sigma_{r}}^{2}\leftarrow {r_{q}}^{2}/12+\;{\alpha_{r}}^{2}\left(1\;-U\right)$
20:              ${\sigma_{\theta }}^{2}\leftarrow \Delta {\theta_{q}}^{2}/12+{\beta_{\theta }}^{2}\;\left(1\;-U\right)$, ${\sigma_{\varphi }}^{2}\leftarrow \Delta {\varphi_{q}}^{2}/12+\;{\beta_{\varphi }}^{2}\left(1\;-U\right)$
21:              $\Sigma_{r\theta \varphi }\leftarrow \;\mathrm{diag}\left({\sigma_{r}}^{2},{\sigma_{\theta }}^{2},{\sigma_{\varphi }}^{2}\right)$, $\Sigma_{xyz,i}\leftarrow J_{\mathrm{cart}}\Sigma_{r\theta \varphi }{J_{\mathrm{cart}}}^{T}$
22:              compute $d_{t},\;\Delta \psi_{t},\;o_{t},\;g_{t}$
23:              $K_{t}\leftarrow (g_{t}+\beta_{1}d_{t}+\beta_{2}\Delta \psi_{t})/(o_{t}+\varepsilon )$
24:              **if** $K_{t}>K_{\mathrm{th}}$ **then**
25:                    insert current frame into K
26:                    $C_{t}\leftarrow \;\left\{k_{i}\in K|0<\Delta t\left(k_{i},t\right)\;\leq \;10\;s\right\}$
27:                    **if** $\left|C_{t}\right|$ is insufficient **then** extend Δt to 20 s
28:                    $M_{t}\leftarrow$ fuse spatially nearest candidates in $C_{t}$
29:                    **for** each $p_{\mathrm{src},i}\in {P_{t}}^{\mathrm{sel}}$**do**
30:                             find correspondence $\left(p_{\mathrm{map},i},n_{i}\right)\;\in M_{t}$
31:                             $e_{i}\leftarrow {n_{i}}^{T}\left(T\left(\xi \right)p_{\mathrm{src},i}-p_{\mathrm{map},i}\right)$
32:                             $w_{\mathrm{cov},i}\leftarrow \;{\left(\zeta +{n_{i}}^{T}R_{\mathrm{src}\to \mathrm{map}}\Sigma_{xyz,i}R_{\mathrm{map}\to \mathrm{src}}n_{i}\right)}^{-1}$
33:                             $w_{i}\leftarrow w_{\mathrm{cov},i}w_{\mathrm{robust},i},\;H_{k}\leftarrow \;\Sigma w_{i}{J_{i}}^{T}J_{i}$
34:                    **end for**
35:                    solve weighted LM for $T\left(\xi \right)$, set $\Sigma_{\mathrm{lidar},k}\leftarrow \;{\left(H_{k}+I\right)}^{-1}$
36:                    add LiDAR pose factor and IMU preintegration factor to G
37:                    $C_{t}^{\mathrm{loop}}\leftarrow \{k_{i}\in K|\Delta t(k_{i},t)>\tau_{t}\}$
38:                    Select $c_{t}$ from $C_{t}^{\mathrm{loop}}$ by overlap and coarse geometric consistency.
39:                    **if** $c_{t}$ is valid **then**
40:                             run weighted point-to-plane ICP between ${P_{t}}^{\mathrm{sel}}$ and $c_{t}$
41:                             obtain $T_{\mathrm{loop}},H_{\mathrm{loop}},\Sigma_{\mathrm{loop}}\leftarrow \;{\left(H_{\mathrm{loop}}+I\right)}^{-1}$
42:                             add loop factor to $G,\;\tilde{l}\leftarrow \;0.5\left(s_{\mathrm{loop}}+s_{\mathrm{icp}}\right)$
43:                    **else**
44:                             $\tilde{l}\leftarrow \;0$
45:                    **end if**
46:                    $l_{t}\leftarrow \;\left(1\;-\tau_{\mathrm{loop}}\right)l_{t-1}+\tau_{\mathrm{loop}}\tilde{l}$
47:                    $\left(\left\{\chi_{k}\right\},{\Sigma_{k}}^{\mathrm{pose}}\right)\;\leftarrow$ iSAM2(G)
48:                    ${\Sigma_{k}}^{xy}$ and $\sigma_{\psi ,k}^{2}$; normalize to ${\mu_{k}}^{p},\;{\mu_{k}}^{\psi }$
49:                    $\mu_{t}\leftarrow \lambda_{p}{\mu_{k}}^{p}+\lambda_{\psi }{\mu_{k}}^{\psi }$
50:              **end if**
51:       **end for**
52:       **return** $\left\{\chi_{k}\right\},\;\mu_{t},\;l_{t}$


## 4. Experiments and Results

In this study, we conducted two comparative experiments on the KITTI dataset [37] and the M2DGR dataset [38], an ablation study, a real-world field test, a sensitivity analysis, and a memory/runtime evaluation to assess the localization accuracy, mapping quality, robustness, and practical efficiency of the proposed method. Furthermore, a real-world field test was conducted to validate the practical robustness of the system. All our implementations were developed in C++ and run on the ROS Melodic framework under Ubuntu 18.04.

### 4.1. Dataset Introduction

KITTI is a widely used multi-sensor benchmark for autonomous driving and mobile robotics. It was collected in Karlsruhe, Germany, and nearby road environments, providing synchronized camera, Velodyne HDL-64 LiDAR, GPS, and IMU data. RTK-corrected GPS/IMU measurements are used as ground-truth references for localization.

M2DGR is a public multi-sensor and multi-scenario SLAM dataset for ground robots. Its platform integrates multiple cameras, an event camera, visual–inertial sensors, IMU, 32-line LiDAR, consumer-grade Global Navigation Satellite System (GNSS), and RTK-enabled GNSS-IMU navigation. All sensors are calibrated and synchronized, with ground-truth trajectories provided by motion capture systems, laser 3D trackers, and RTK technology.

### 4.2. Experimental Setup

In the comparative experiments, we adopted the Root-Mean-Square Error (RMSE) and maximum error of the absolute trajectory error (ATE) as the evaluation metrics. The RMSE measures the average magnitude of deviation between the estimated trajectory and the ground truth, providing a quantitative assessment of the system’s localization accuracy, while the maximum error captures the worst-case deviation occurring throughout the algorithm’s operation, indicating the upper bound of localization error under extreme conditions. In the comparative experiment tables, the localization results of the best-performing algorithm on each dataset are highlighted in bold for clearer visual comparison.

To ensure reproducibility, all experiments used a unified parameter setting unless otherwise stated. The base depth image resolution was set to 64×1800 for the KITTI data and to 32×1800 for the 32-line LiDAR data, with the adaptive coefficient limited to κ∈[0.80,1.35] and the following feedback gains: $k_{u}=0.25,k_{l}=0.20$. The depth image was divided into 4×8 pixel blocks. The four normalized scores for density, depth reliability, geometric complexity, and motion consistency were weighted as 0.25, 0.25, 0.30, and 0.20, with $\lambda_{u}=0.20$ and $\lambda_{l}=0.15$. The retention threshold ($U_{\mathrm{th}}$) was set as the 80th percentile of block scores and clipped to [0.55,0.70], retaining about 20% of non-ground points. For motion perception, γ=2, and the effective range was 5–60 m. Pose uncertainty was normalized by 0.25 m for planar translation and by $5^{{}^{\circ}}$ for yaw and then fused with $\lambda_{p}=0.70$ and $\lambda_{\psi }=0.30$. The covariance coefficients were $\alpha_{r}=0.03$, $\beta_{\theta }=0.002$, $\beta_{\phi }=0.004$ and $\zeta =10^{-6}$. For keyframe and loop processing, $K_{\mathrm{th}}=0.30$, $\beta_{1}=0.50{\;m}^{-1}$, $\beta_{2}=1.00{\;\mathrm{rad}}^{-1}$ and $\epsilon =10^{-3}$; the local-map window was 10–20 s; loop candidates within 30 s were excluded; and $\tau_{\mathrm{loop}}=0.20$. The default Huber threshold ($\delta_{\mathrm{Huber}}$) was set to 0.15 m. For the compared methods, we used their publicly available open-source implementations and kept the default parameters recommended by the original authors.

### 4.3. Comparison of Mapping Performances

**Figure 8:**
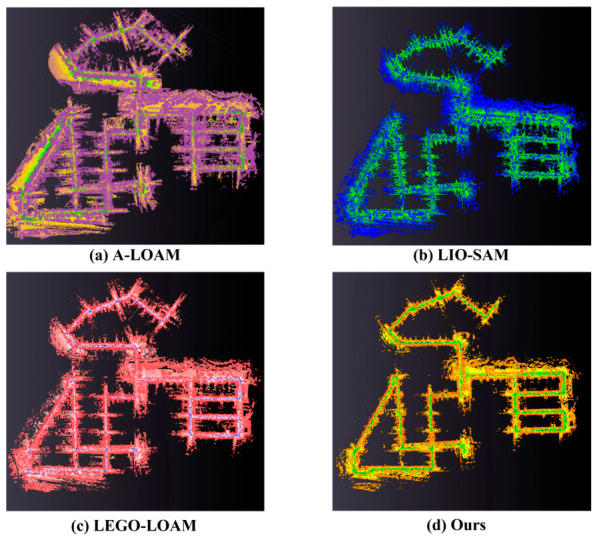
Comparative mapping results.

To validate the practical mapping performance of the proposed method and assess its ability to reconstruct clean and detailed large-scale scenes, we evaluated the mapping quality of our proposed method against that of A-LOAM, LIO-SAM, and LEGO-LOAM on sequence 08 of the KITTI dataset. The comparative results are presented in Figure 8. To facilitate visual comparison, the map generated by each algorithm is rendered in a distinct color.

As clearly demonstrated, the global map generated by our method not only reconstructs the urban landscape more accurately but also contains significantly less noise. This superior performance can be attributed to two main factors. First, our method achieved higher localization accuracy on this dataset compared to the other three approaches. Second, our novel adaptive depth image construction, coupled with a scoring mechanism that prioritizes pixels with distinctive observational features, enables the reconstruction of cleaner and higher-quality point clouds for map building.

**Figure 9:**
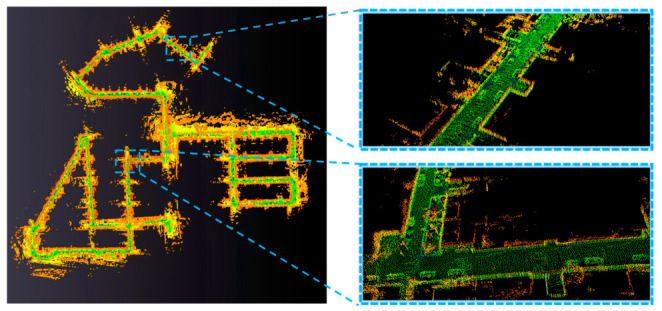
Detailed visualization of proposed method’s mapping results.

Furthermore, our method excels at preserving fine-grained structural details of the physical world. Figure 9 illustrates two specific scenes extracted from the global map. In these selected real-road scenarios, our constructed map clearly delineates road contours and captures the distinct shapes of vehicles, showcasing its capability to recover rich environmental details.

### 4.4. Comparison on KITTI Dataset

**Figure 10:**
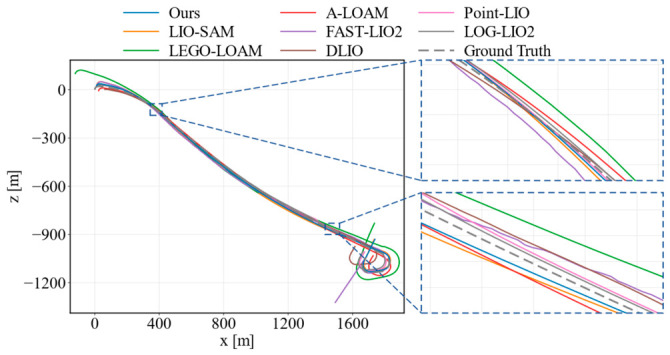
Trajectory comparison on KITTI sequence 01.

**Figure 11:**
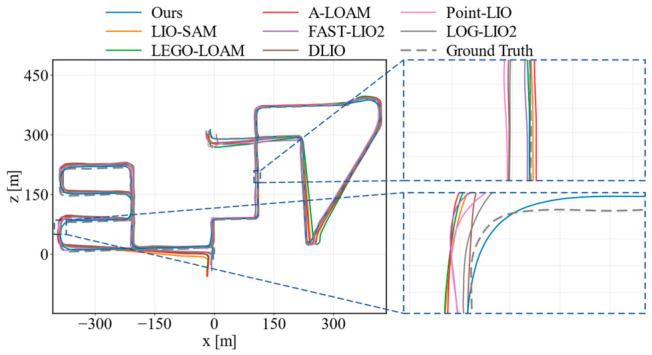
Trajectory comparison on KITTI sequence 08.

**Figure 12:**
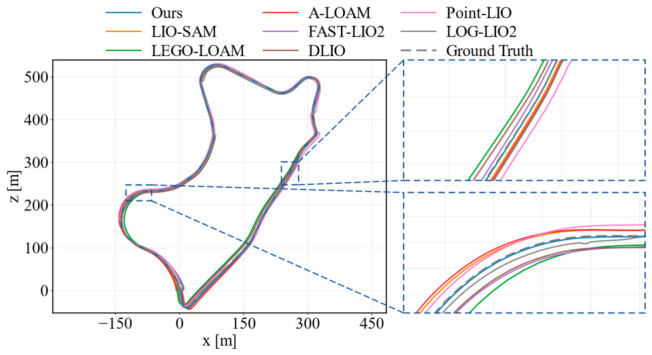
Trajectory comparison on KITTI sequence 09.

The localization accuracy of the proposed method was evaluated against those of seven representative LiDAR or LiDAR–inertial SLAM methods, A-LOAM, LEGO-LOAM, LIO-SAM, FAST-LIO2, DLIO, LOG-LIO2, and Point-LIO, across seven KITTI sequences. These baselines cover feature-based LiDAR odometry, graph-based LiDAR–inertial smoothing, direct LiDAR–inertial odometry, and recent point-wise or loop-enhanced LIO pipelines. Therefore, the comparison provides a broad evaluation under different algorithmic assumptions. The quantitative results are summarized in Table 1, and representative trajectory comparisons are shown in Figure 10, Figure 11 and Figure 12.

As shown in Table 1, the proposed method achieved the lowest average RMSE and the lowest average maximum ATE error among all compared methods, indicating a stable overall localization performance on the KITTI dataset. In terms of the RMSE, our method obtained the best results on sequences 00, 08, and 09 while remaining close to the best-performing baseline on sequences 01, 04, 06, and 07. For the maximum error metric, our method also shows the lowest error on sequences 00, 01, 08, and 09, suggesting that it can effectively suppress large trajectory deviations in long-distance driving scenarios. Although some baselines performed slightly better on individual sequences, their errors are less consistent across the full benchmark. In contrast, the proposed method maintains a better balance between average accuracy and worst-case robustness.

Figure 10, Figure 11 and Figure 12 further compare the estimated trajectories on KITTI sequences 01, 08, and 09. Two enlarged regions are provided in each figure to highlight local alignment differences. On sequence 01, FAST-LIO2 gives a slightly lower RMSE, but the trajectory of our method remains close to the ground truth and shows competitive local consistency in the enlarged regions. On sequence 08, our method follows the ground-truth trajectory more closely in both the global view and local details, which is consistent with its lowest RMSE and maximum error on this sequence. On sequence 09, the proposed method also presents smaller deviations in curved and transition regions compared with most baselines. These results show that the proposed feedback-driven point selection and uncertainty-aware optimization can improve trajectory consistency while reducing accumulated drift in urban driving environments.

### 4.5. Comparison on M2DGR Dataset

**Figure 13:**
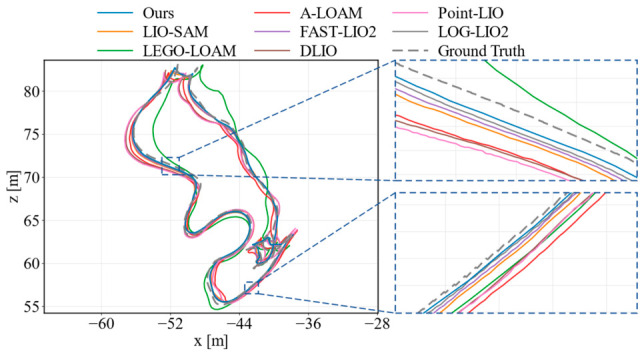
Trajectory comparison on M2DGR gate_01.

**Figure 14:**
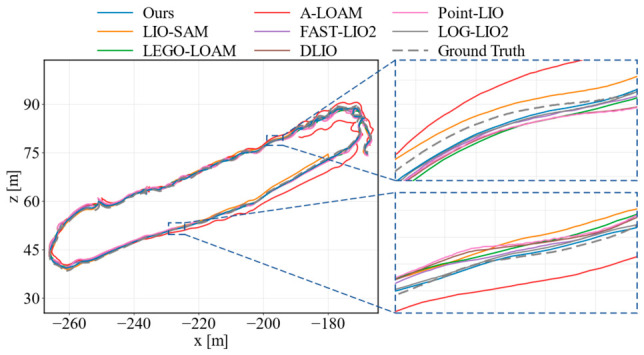
Trajectory comparison on M2DGR street_08.

To further evaluate the generality of the proposed method on ground-robot data, we conducted comparative experiments on four representative M2DGR sequences, including gate_01, street_04, street_05, and street_08. The compared methods were the same as those used in the KITTI evaluation, covering both LiDAR-only and LiDAR–inertial SLAM pipelines. The quantitative results in terms of the RMSE and maximum ATE error are reported in Table 2. Figure 13 and Figure 14 show the trajectory comparisons on gate_01 and street_08, where two enlarged regions are provided to illustrate local alignment differences.

As shown in Table 2, the proposed method achieved the lowest average RMSE among all compared methods on the selected M2DGR sequences. It obtained the best RMSE on gate_01 and street_08 while remaining competitive on street_04 and street_05. In terms of the maximum error, our method achieved the lowest value on gate_01 and the second-lowest value on street_08, although LOG-LIO2 performed slightly better in terms of the average maximum error. These results indicate that the proposed method does not rely on a single dataset setting and can maintain stable accuracy on ground-robot sequences with different motion patterns and scene structures.

Figure 13 shows the gate_01 sequence, which contains continuous turns and irregular loop-like motion. In the global trajectory and the enlarged regions, A-LOAM, LEGO-LOAM, and Point-LIO show more visible offsets from the reference trajectory, especially around curved segments. By contrast, the proposed method keeps closer local alignment during these maneuvers, which is consistent with its lowest RMSE and maximum error on this sequence. Figure 14 presents the longer street_08 sequence. The main difference appears in the two enlarged regions, where A-LOAM and LIO-SAM exhibit larger local deviations, while our method remains close to the reference trajectory and comparable to the strongest recent baselines. Overall, the M2DGR results provide complementary evidence that the feedback-guided point selection and uncertainty-aware optimization improve trajectory consistency beyond the KITTI driving scenarios.

### 4.6. Ablation Study

To further validate the contribution of each key component in our system, we conducted an ablation study and report the results. As summarized in Table 3, these variants separately disable backend feedback, adaptive resolution, score-based point selection, quantization-based covariance weighting, and score-based covariance weighting. Ours-NF disables backend feedback. Since the adaptive resolution is driven by the feedback scalars, the depth image resolution is fixed to the base setting in this variant, while score-based selection and covariance weighting are retained. In Ours-US, the frontend score is still calculated for covariance weighting, but it is not used for point selection. The retained points are selected uniformly with a similar retention ratio.

As shown in Table 4, the full method achieved the lowest RMSE and maximum ATE error on all evaluated sequences, indicating that the proposed components are complementary. Compared with Ours-Base, the full method reduced the mean RMSE from 8.380 m to 5.784 m, showing that the improvement was not only caused by the shared backend framework. Ours-NF and Ours-FR both degraded performance, which confirms the usefulness of backend feedback and adaptive resolution. The larger degradation of Ours-US indicates that score-based point selection plays an important role in retaining informative observations. In addition, Ours outperformed Ours-Q, Ours-S, and Ours-FW, suggesting that the quantization error and frontend score provide complementary cues for covariance weighting.

### 4.7. Field Test

**Figure 15:**
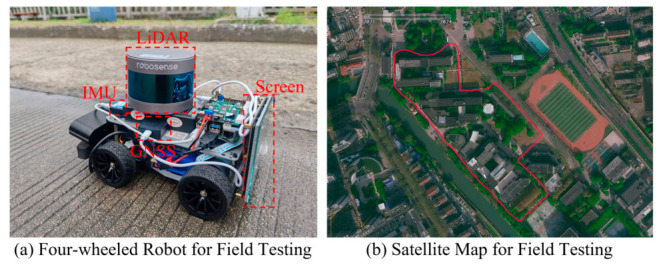
Experimental setup and testing environment for real-world field evaluation.

To further validate the practical robustness and generalization ability of the proposed method in real-world scenarios, a field test was conducted. As shown in Figure 15, data was collected in a complex campus environment using a four-wheeled mobile robot equipped with a 360-degree rotating LiDAR, an IMU, and a GNSS.

In the complex campus environment, centimeter-level external ground truth was unavailable because the compact GNSS receiver was affected by tree canopies, surrounding buildings, and multipath effects. Therefore, we constructed an approximate multi-source reference trajectory for comparative evaluation. Before data collection, 40 visible control markers were placed along the campus loop, with the same physical marker used as both the start and end point. A local 2D coordinate system was defined using this marker as the origin and the initial driving direction as the positive *x*-axis. The remaining marker positions were surveyed using a handheld laser rangefinder, a measuring tape, and route geometry constraints. During data collection, the robot was remotely controlled to pass these markers sequentially, and the corresponding timestamps were recorded. The raw GNSS/IMU trajectory provided a continuous coarse trajectory and scale reference, while the measured marker positions and the same-marker start-to-end loop closure constraint provided stronger geometric constraints. These constraints were fused offline using pose graph smoothing with a standard nonlinear least-squares solver.

To estimate the reliability of the constructed reference, we calculated the residuals between the smoothed trajectory and the 40 control markers. The marker residuals had an RMSE of approximately 0.22 m, a 95th-percentile error of about 0.38 m, and a maximum local residual of 0.51 m, mainly near tree-covered or turning segments. The start-to-end discrepancy at the repeated physical marker was reduced to 0.08 m after loop closure smoothing. This reference trajectory was used only for post-evaluation and was not involved in the frontend point cloud selection, scan-to-map registration, loop closure detection, or factor graph optimization of any tested SLAM method. Therefore, the field test ATE values should be interpreted as approximate comparative indicators under real-world operating conditions rather than as centimeter-level absolute localization errors.

**Figure 16:**
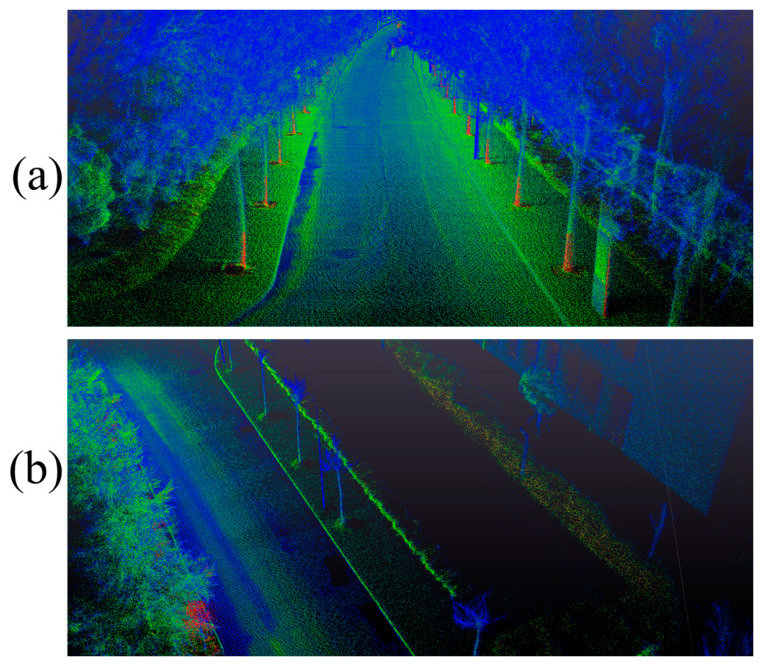
3D point cloud mapping performance of the proposed method in the field test: (**a**) tree-lined road scene; (**b**) roadside/building scene.

Figure 16 visually demonstrates the 3D point cloud mapping performance in two different environmental scenarios. The proposed method successfully reconstructs highly consistent and dense maps, accurately capturing fine structural details, such as tree canopies and road boundaries, without noticeable ghosting effects.

**Figure 17:**
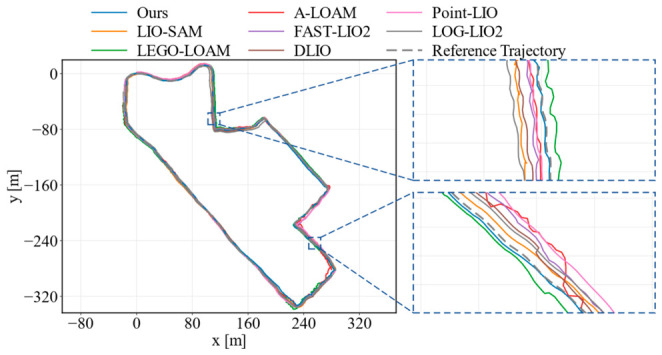
Trajectory comparison of different SLAM algorithms in real-world field test.

The quantitative and visual ATE results with respect to the constructed field reference trajectory are summarized in Table 5 and Figure 17. The proposed method achieved the lowest RMSE of 1.591 m, the lowest median error of 1.003 m, and the lowest maximum error of 4.313 m among all compared methods, indicating a more stable overall performance during the campus loop. Although some baselines obtained slightly smaller minimum errors at isolated locations, their RMSEs and median and maximum errors are higher, suggesting less consistent trajectory accuracy over the full route. Compared with FAST-LIO2 and LIO-SAM, the proposed method reduced the RMSE by 26.3% and 35.7%, respectively. In Figure 17, all tested methods complete the large-scale loop, but the enlarged regions show clearer local differences: several baselines exhibit noticeable offsets during continuous turns and along the oblique road segment, whereas the proposed method remains closer to the reference trajectory. These results suggest that the proposed feedback-driven point selection and uncertainty-aware optimization can improve drift suppression and trajectory consistency under the tested real-world conditions.

### 4.8. Sensitivity Analysis Experiment

To further verify the reliability of the proposed covariance-guided weighting and the robustness of the key parameters, we conducted a sensitivity analysis experiment. First, using KITTI sequence 08 and M2DGR street_08 as representative driving and ground-robot sequences, we collected the covariance weight ($w_{\mathrm{cov},i}$) and corresponding absolute point-to-plane residual ($\left|e_{i}\right|$) after scan-to-map ICP convergence to examine whether the proposed weight was correlated with actual registration reliability. Then, we tested three representative parameter groups: the covariance weighting strength, the Huber kernel threshold ($\delta_{\mathrm{Huber}}$), and the keyframe selection threshold ($K_{\mathrm{th}}$). The covariance weighting strength is controlled by $\lambda_{\mathrm{cov}}$, which is defined as a common multiplier applied to the covariance coefficients $\alpha_{r}^{2}$, $\beta_{\theta }^{2}$, and $\beta_{\phi }^{2}$. The default setting corresponds to $\lambda_{\mathrm{cov}}=1.0$. For the sensitivity analysis, $\lambda_{\mathrm{cov}}$, $\delta_{\mathrm{Huber}}$, and $K_{\mathrm{th}}$ were varied using multipliers of 0.5×, 0.75×, 1.0×, 1.25×, and 1.5×, while all other parameters were kept unchanged.

**Figure 18:**
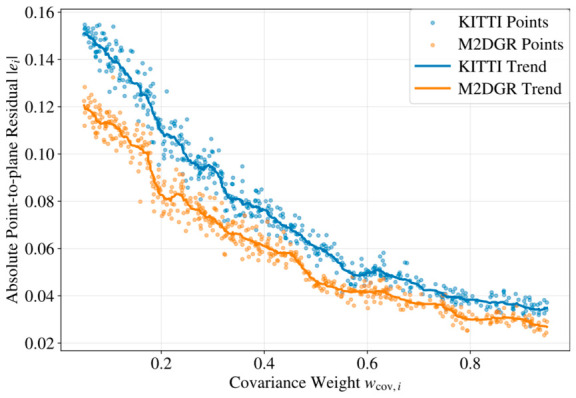
Relationship between covariance weight and point-to-plane residual on KITTI sequence 08 and M2DGR street_08.

**Figure 19:**
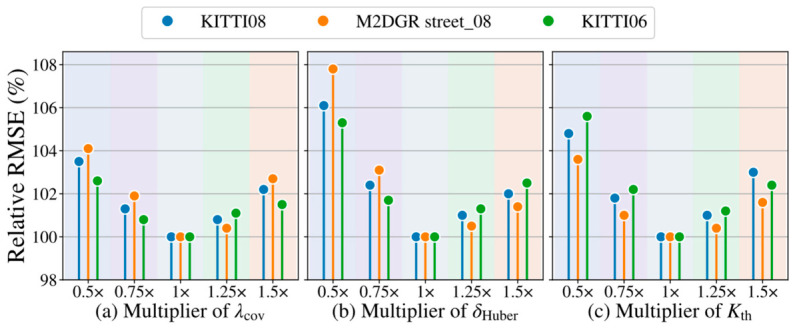
Sensitivity analysis of three representative parameters.

As shown in Figure 18, larger covariance weights generally correspond to smaller point-to-plane residuals, indicating that the proposed covariance-guided weight can provide a meaningful reliability cue for scan-to-map registration. Figure 19 further shows that the default settings achieved the best or near-best performance for all three tested parameters. Among them, $\delta_{\mathrm{Huber}}$ has the most obvious influence, since an overly small threshold may suppress valid residuals, while an overly large threshold weakens outlier rejection. In comparison, $\lambda_{\mathrm{cov}}$ and $K_{\mathrm{th}}$ show moderate sensitivity, and the performance remains stable within a reasonable range around 1.0×. These results suggest that the proposed method does not rely on overly delicate parameter tuning and has good robustness under moderate parameter variations.

### 4.9. Memory and Runtime Test

To evaluate the practical efficiency of the proposed method, a memory and runtime test was conducted against two representative LiDAR–inertial SLAM baselines, FAST-LIO2 and LIO-SAM. All methods were tested under the same hardware and software environment using KITTI sequence 08 as the input sequence, and the per-frame runtime and memory consumption were recorded over 184 consecutive frames. The runtime reflects the overall computational cost of each SLAM pipeline, while the memory consumption reflects the storage burden caused by point cloud processing, local map maintenance, and backend optimization.

**Figure 20:**
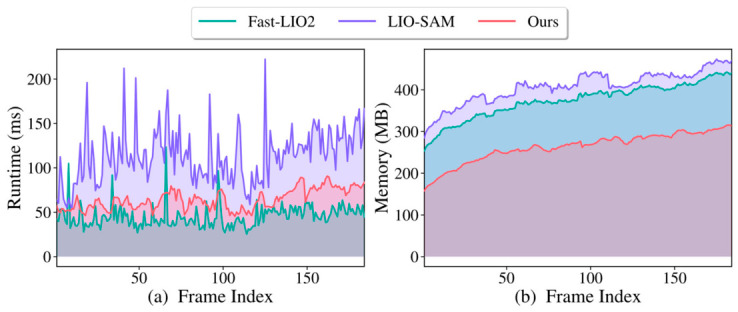
Runtime and memory comparison of FAST-LIO2, LIO-SAM, and proposed method over consecutive frames.

As shown in Figure 20a, FAST-LIO2 achieved the lowest average runtime of 45.46 ms per frame, mainly because it adopts an iterated Kalman filtering framework and directly registers raw points to the map without explicit feature extraction or graph-based smoothing. LIO-SAM has the highest average runtime of 113.59 ms per frame and shows larger fluctuations, mainly caused by feature extraction, keyframe-based local map construction, scan-to-map matching, IMU preintegration, and incremental factor graph updates. The proposed method achieved an average runtime of 64.39 ms per frame. Although it introduces adaptive point selection, covariance weighting, loop processing, and factor graph optimization, the compact high-quality point subset reduces the cost of scan-to-map matching and map maintenance, keeping the runtime clearly lower than that of LIO-SAM. Figure 20b further shows that the proposed method maintained the lowest memory consumption, with an average of 263.21 MB, compared with 374.92 MB for FAST-LIO2 and 408.13 MB for LIO-SAM. This reduction is mainly attributed to retaining about 20% of the non-ground points for registration and map updating. Overall, the proposed method provides a practical balance between localization accuracy, runtime efficiency, and memory usage.

## 5. Discussion

The experimental results indicate that the proposed framework benefits from the coordinated design of frontend point cloud regulation and backend reliability-aware optimization. On KITTI and M2DGR, the method achieved the lowest average RMSE among the tested LiDAR and LiDAR–inertial SLAM methods while also maintaining a competitive maximum error performance. The mapping results show clearer road boundaries and object structures, suggesting that retaining compact but informative points can improve both localization and map quality. In the campus field test, the proposed method also obtained the lowest RMSE with respect to the constructed reference trajectory, indicating practical applicability in a complex outdoor environment.

The ablation and sensitivity results further show that the performance gain does not come from a single module alone. Backend feedback helps regulate depth image resolution and point cloud retention, score-based selection removes redundant or unstable observations before registration, and covariance weighting assigns different reliabilities to retained points during scan-to-map ICP and factor graph optimization. The finer ablation variants show that feedback, adaptive resolution, point selection, quantization-based weighting, and score-based weighting all contribute to different degrees. The sensitivity analysis also indicates that larger covariance-guided weights generally correspond to smaller point-to-plane residuals, and that the performance remains stable under moderate parameter variations. Meanwhile, the runtime and memory results show that the proposed method reduces memory consumption while keeping a moderate computational cost, mainly due to the sparse frontend representation.

Several limitations remain. First, although the sensitivity analysis shows moderate robustness around the default settings, some thresholds, weights, and normalization constants are still empirically selected and may require recalibration for different LiDAR beam patterns, motion regimes, or non-forward-dominant platforms. Second, the covariance used in this work should be interpreted as a practical observation reliability weight derived from projection quantization and point quality rather than as a complete physical sensor noise model. In addition, the proposed method does not explicitly segment or remove dynamic objects. Dynamic measurements are only indirectly down-weighted when they produce unstable point quality scores, larger covariance-derived variances, or large scan-to-map residuals under the Huber kernel. Therefore, highly dynamic scenes with many independently moving objects remain challenging and require further dedicated evaluation. Third, the feedback variables are compressed into scalar indicators for online efficiency, which cannot fully describe multi-directional pose uncertainty. Finally, because the field test reference trajectory was constructed through multi-source measurements, marker constraints, loop closure, and offline smoothing, the corresponding ATE values should be regarded as approximate comparative indicators rather than as laboratory-grade absolute ground truth. Future work will focus on automatic parameter tuning, richer uncertainty feedback, and more rigorous field reference trajectory construction for broader real-world evaluations.

## 6. Conclusions

In this paper, we propose a feedback-coupled LiDAR–inertial SLAM framework that connects frontend point cloud processing and backend pose optimization through reliability-related information exchange. The backend estimates pose uncertainty and loop closure importance to regulate frontend depth image resolution and point cloud retention. The frontend then combines point quality with projection quantization error to construct observation covariance weights for scan-to-map ICP and factor graph optimization. This design allows the system to retain compact but informative observations and to weight them according to their estimated reliability.

Experiments on KITTI and M2DGR and a campus field test show that the proposed method achieved competitive and generally improved localization accuracy under the evaluated conditions. Compared with representative LiDAR-only and LiDAR–inertial baselines, including feature-based, graph-based, direct, and point-wise LIO methods, the proposed method obtained the lowest average RMSE on both benchmark datasets. The ablation study supports the contribution of the feedback mechanism, adaptive resolution, score-based point selection, and covariance-weighted optimization. The sensitivity analysis further shows that the proposed weighting strategy provides a useful reliability cue, and that the system is not overly sensitive to moderate parameter variations. The runtime and memory results also indicate that the frontend point selection reduces memory usage while maintaining reasonable computational cost.

The field experiment demonstrates the practical applicability of the framework in a complex campus environment, where the proposed method produced consistent mapping results and lower trajectory error with respect to the constructed reference trajectory. Nevertheless, the current method still relies on empirically selected parameters and scalar feedback variables, and the field test reference remains an approximate evaluation reference. Future work will aim to reduce manual parameter tuning, introduce richer uncertainty representations, and evaluate the framework under more diverse sensors, motion patterns, sparse scenes, and dynamic environments.

## Figures and Tables

**Table 1:** RMSEs (m) and maximum ATE errors (m) on KITTI dataset.

Method	Seq 00	Seq 01	Seq 04	Seq 06	Seq 07	Seq 08	Seq 09	Mean
RMSE (m)								
Ours	**5.964**	16.782	0.316	2.239	0.698	**18.315**	**3.242**	**6.794**
A-LOAM	11.482	34.974	0.385	4.812	0.729	34.438	4.307	13.018
LEGO-LOAM	8.678	156.599	0.564	**2.236**	3.598	30.509	6.225	29.772
LIO-SAM	7.339	19.768	**0.298**	2.901	0.733	28.553	3.863	9.065
FAST-LIO2	6.546	**16.537**	0.441	3.407	0.624	23.329	5.573	8.065
DLIO	6.887	69.891	0.413	4.239	0.782	33.467	4.818	17.214
LOG-LIO2	6.218	30.532	0.472	3.711	**0.573**	29.117	3.915	10.648
Point-LIO	7.317	25.129	0.566	4.059	0.681	26.248	5.151	9.879
MAX ERROR (m)								
Ours	**9.065**	**24.195**	0.745	4.723	1.033	**27.411**	**6.122**	**10.471**
A-LOAM	27.671	85.525	0.854	11.452	1.786	60.745	8.345	28.054
LEGO-LOAM	20.393	313.194	1.212	**4.341**	6.032	53.042	9.454	58.238
LIO-SAM	14.311	38.148	0.781	5.482	1.407	60.646	8.233	18.429
FAST-LIO2	12.306	34.195	0.798	6.541	1.162	52.352	11.501	16.979
DLIO	13.154	97.286	0.764	8.308	1.509	50.375	8.997	25.771
LOG-LIO2	11.503	42.736	**0.643**	7.088	**0.894**	42.912	7.123	16.128
Point-LIO	14.487	36.912	1.032	7.592	1.283	36.718	8.222	15.178

**Table 2:** RMSEs (m) and maximum ATE errors (m) on M2DGR dataset.

Method	Gate_01	Street_04	Street_05	Street_08	Mean
RMSE (m)					
Ours	**0.647**	1.265	0.752	**0.872**	**0.884**
A-LOAM	1.789	3.558	0.828	3.168	2.336
LEGO-LOAM	2.823	1.524	0.963	2.008	1.830
LIO-SAM	0.982	2.866	**0.613**	1.956	1.604
FAST-LIO2	1.137	1.046	0.744	1.243	1.043
DLIO	2.287	1.257	1.073	2.777	1.849
LOG-LIO2	0.873	**0.987**	0.953	0.989	0.951
Point-LIO	2.465	1.101	0.877	2.914	1.839
MAX ERROR (m)					
Ours	**0.926**	2.887	1.164	1.507	1.621
A-LOAM	3.054	5.231	1.739	5.347	3.843
LEGO-LOAM	5.212	2.593	1.733	3.102	3.160
LIO-SAM	1.579	4.156	**0.923**	2.502	2.290
FAST-LIO2	1.795	1.937	1.562	3.294	2.147
DLIO	3.642	2.075	1.878	4.669	3.066
LOG-LIO2	1.190	**1.579**	1.324	**1.499**	**1.398**
Point-LIO	3.872	1.762	1.493	4.869	2.999

**Table 3:** Configurations of ablation variants.

Method	Feedback	Adaptive Resolution	Score-Based Selection	Quantization Term	Score Term
Ours	✓	✓	✓	✓	✓
Ours-Base	–	–	–	–	–
Ours-NF	–	–	✓	✓	✓
Ours-FR	✓	–	✓	✓	✓
Ours-US	✓	✓	–	✓	✓
Ours-Q	✓	✓	✓	✓	–
Ours-S	✓	✓	✓	–	✓
Ours-FW	✓	✓	✓	–	–
Ours-CH	✓	✓	✓	✓	✓
Ours-TK	✓	✓	✓	✓	✓

Note: ✓ indicates that the component is enabled, and–indicates that it is disabled. Ours-CH and Ours-TK only replace the Huber kernel in Ours with the Cauchy and Tukey kernels.

**Table 4:** RMSE (m) and MAX ERROR (m) comparison of ATE accuracy results of ablation study.

Method	KITTI_01	KITTI_06	KITTI_09	Street_08	Mean
RMSE (m)					
Ours	16.782	2.239	3.242	0.872	5.784
Ours-Base	23.041	3.018	5.764	1.695	8.380
Ours-NF	18.936	2.581	4.164	1.157	6.710
Ours-FR	17.692	2.468	3.781	1.042	6.246
Ours-US	20.185	2.673	4.506	1.318	7.170
Ours-Q	18.347	2.512	3.936	1.216	6.503
Ours-S	18.982	2.604	4.391	1.268	6.811
Ours-FW	21.873	2.851	5.332	1.611	7.917
Ours-CH	16.934	2.386	3.358	0.985	5.916
Ours-TK	17.126	2.318	3.291	1.013	5.937
MAX ERROR (m)					
Ours	24.195	3.023	5.922	1.507	8.662
Ours-Base	32.418	4.183	9.576	2.514	12.173
Ours-NF	26.042	3.452	6.836	1.630	9.490
Ours-FR	25.018	3.386	6.527	1.566	9.124
Ours-US	28.714	3.742	7.389	1.945	10.447
Ours-Q	26.375	3.291	6.642	1.793	9.525
Ours-S	27.004	3.587	7.118	1.846	9.889
Ours-FW	30.872	4.044	8.926	2.427	11.567
Ours-CH	24.584	3.097	6.108	1.544	8.833
Ours-TK	25.012	3.154	6.031	1.573	8.943

**Table 5:** RMSEs and minimum, median, and maximum ATE errors (m) in field test.

Method	RMSE (m)	MIN ERROR(m)	MEDIAN ERROR(m)	MAX ERROR (m)
Ours	**1.591**	0.612	**1.003**	**4.313**
A-LOAM	3.859	0.778	1.374	5.714
LEGO-LOAM	3.417	0.507	1.926	5.384
LIO-SAM	2.475	0.654	1.179	5.161
FAST-LIO2	2.158	**0.428**	1.256	5.708
DLIO	3.116	0.662	1.622	5.819
LOG-LIO2	2.681	0.631	1.123	5.201
Point-LIO	2.552	0.602	1.437	5.423

## Data Availability

The original contributions presented in this study are included in the article. Further inquiries can be directed to the corresponding author.
